# Evaluation of *in vivo* responses of sorafenib therapy in a preclinical mouse model of *PTEN*-deficient of prostate cancer

**DOI:** 10.1186/s12967-015-0509-x

**Published:** 2015-05-08

**Authors:** Yutaka Yamamoto, Marco A De Velasco, Yurie Kura, Masahiro Nozawa, Yuji Hatanaka, Takashi Oki, Takayuki Ozeki, Nobutaka Shimizu, Takafumi Minami, Kazuhiro Yoshimura, Kazuhiro Yoshikawa, Kazuto Nishio, Hirotsugu Uemura

**Affiliations:** Department of Urology, Kinki University Faculty of Medicine, 377-2 Ohno-Higashi, Osaka-Sayama, 589-8511 Japan; Department of Genome Biology, Kinki University Faculty of Medicine, Osaka-Sayama, 589-8511 Japan; Promoting Center for Clinical Research, Aichi Medical University, School of Medicine, Nagakute, Aichi 480-1195 Japan

**Keywords:** Prostate cancer, Castration-resistant prostate cancer, PTEN, Combination therapy, Sorafenib, Everolimus, Mouse model, Preclinical

## Abstract

**Background:**

Despite recent advances in the treatment for advanced prostate cancer, outcomes remain poor. This lack of efficacy has prompted the development of alternative treatment strategies. In the present study we investigate the effects of the multikinase inhibitor sorafenib in a genetically engineered mouse model of prostate cancer and explore the rational combination with the mTOR inhibitor everolimus.

**Methods:**

Conditional prostate specific *PTEN*-deficient knockout mice were utilized to determine the pharmacodynamic and chemopreventive effects of sorafenib. This mouse model was also used to examine the therapeutic efficacy of sorafenib alone or in combination with everolimus. Preclinical efficacy was assessed by comparing the reduction of tumor burden, proliferation, angiogenesis and the induction of apoptosis. Molecular responses were assessed by immunohistochemical, TUNEL and western blot assays.

**Results:**

Pharmacodynamic analysis revealed that a single dose of sorafenib decreased activation of the PI3K/AKT/mTOR signaling axis at doses of 30–60 mg/kg, but activated JAK/STAT3 signaling. Levels of cleaved casapase-3 increased in a dose dependent manner. Chemoprevention studies showed that chronic sorafenib administration was capable of inhibiting tumor progression through the reduction of cancer cell proliferation, angiogenesis and the induction of apoptosis. In intervention models of established castration-naïve and castration-resistant prostate cancer, treatment with sorafenib provided modest but statistically insignificant reduction in tumor burden. However, sorafenib significantly inhibited cancer cell proliferation and MVD but had minimal effects on the induction of apoptosis. Interestingly, the administration of sorafenib increased the expression levels of the androgen receptor, p-GSK3β and p-ERK1/2 in castration-resistant prostate cancers. In both intervention models, combination therapy demonstrated a clear tendency of enhanced antitumor effects over monotherapy. Notably, the treatment combination of sorafenib and everolimus overcame therapeutic escape from single agent therapy in castration-resistant prostate cancers.

**Conclusions:**

In summary, we provide insights into the molecular responses of sorafenib therapy in a clinically relevant model of prostate cancer and present preclinical evidence for the development of targeted treatment strategies based on the use of multikinase inhibitors in combination with mTOR inhibitors for the treatment of advanced prostate cancer.

**Electronic supplementary material:**

The online version of this article (doi:10.1186/s12967-015-0509-x) contains supplementary material, which is available to authorized users.

## Background

Strides have been made for the treatment of localized prostate cancer, including surgery and radiotherapy, however, androgen deprivation therapy (ADT) remains the standard treatment of advanced prostate cancer. Even though tumors initially respond to ADT, most patients invariably develop lethal castration-resistant prostate cancer (CRPC) within only a few years after the initiation of ADT [1-3]. Several new agents such as docetaxel, cabazitaxel, abiraterone acetate, enzalutamide and sipuleucel-T have shown some clinical improvements for CRPC patients, however, responses are modest and the median increase in survival remains poor [4-8]. Thus, novel treatment strategies that could prevent disease progression in CRPC are needed. Recently, a number of small molecules targeting kinases have been developed and have been or are undergoing clinical evaluation. Kinases have the potential to be effective targets for anticancer therapy since these modulate a number of signal transduction cascades. Moreover, many of these kinases play roles in modulating transformed malignant cells as well as non-malignant cells in the tumor microenvironment.

Sorafenib is an oral multi-targeted kinase inhibitor that has demonstrated the ability to suppress Raf kinases as well as a number of receptor tyrosine kinases (RTK) implicated in tumor progression and angiogenesis [9]. Sorafenib has been shown to act directly on human prostate cancer cells decreasing cellular proliferation and inducing apoptosis through the downregulation of AKT and androgen receptor pathways [10]. Moreover sorafenib has been shown to inhibit the activity of full length AR and AR lacking the AR-ligand binding domain in CRPC cells [11]. However, neither sorafenib nor sunitinib, another multi-targeted RTK inhibitor, have lived up to their promise as single agent therapy having shown only modest clinical benefits in human CRPC [12-17]. This lack of efficacy suggests that other pathways are involved in the survival and growth of tumors. In addition, treatment with sorafenib can lead to the activation of c-Met and mTOR in some cell types [18].

The PI3K/AKT/mTOR pathway plays a crucial role in the development of prostate cancer and transformation to CRPC. In metastatic prostate cancer, alterations of the PI3K/AKT/mTOR pathway have been reported in 100% of cases [19]. These alterations occur as a result of AKT activation due to loss of function of the tumor suppressor phosphatase and tensin homolog (*PTEN)* [20]*.* As a result, a great deal of interest has been focused in developing therapeutic agents targeting the PI3K/AKT/mTOR signaling axis. Due to their promising results in preclinical models, several rapalogs, including everolimus, have been tested in the clinical setting but have met disappointing outcomes [17,21-23]. Recent studies have shown that in certain cancer cell types, combining sorafenib with mTOR inhibitors can overcome therapeutic escape [18,24-29].

In this study, we utilize a genetically engineered mouse model of *PTEN*-deficient prostate cancer to characterize the effects of sorafenib on castration-naïve and castration-resistant prostate tumors. We also utilize this mouse model to explore the efficacy of combination therapy with sorafenib and the mTOR inhibitor everolimus.

## Material and methods

### Reagents and antibodies

Sorafenib tysolate and everolimus were purchased form L.C. Laboratories (Woburn, MA). For all *in vivo* studies, sorafenib and everolimus were prepared as previously described [9,30]. Antibodies for western blot and immunohistochemical analysis were purchased as follows: β-catenin (#8480), Bcl-2 (#2870), BIM (#2933), cleaved caspase-3 (#9661), ERK (#9102), GAPDH (#2118), mTOR (#2983), p-β-catenin (#9561), p-ERK (#4370), p-mTOR (#2971), p-S6 (#2211), p-STAT3-pY705 (#9145), p-STAT3-pY727 (#9134), S6 (#2217) and STAT3 (#4904) antibodies from Cell Signaling Technology (Danvers, MA); AR (#RB-9030), PCNA (#RB-9055) and Ki67 (#RB-9043) antibodies from Thermo Scientific (Waltham, MA); cyclin B1 (sc-752), cyclin D1 (sc-753) antibodies from Santa Cruz Biotechnology (Dallas, TX); CD31 (#550300) from BD Biosciences (San Jose, CA).

### Animals

Homozygous *PTEN*-conditional knockout (*PSA*^*Cre*^*/PTEN*^*Flox/Flox*^) mice were used for this study. The characterization of this animal model has been described previously [30]. Briefly, we used the *PSA-Cre* promoter to mediate Cre-lox recombination of exons 4 and 5 (exon 5 encodes the crucial phosphatase core motif). This results in the prostate-specific inactivation of *PTEN* in the dorsolateral and ventral lobes of the prostate. In homozygous mutants, *PSA-Cre* driven inactivation of *PTEN* leads to the development of mouse low grade prostatic intraepithelial neoplasia (lgPIN) at 8 weeks of age which progresses to mouse high grade PIN (hgPIN, a precursor to invasive adenocarcinoma) by 15 weeks of age. To induce CRPC, homozygous *PTEN*-conditional knockout mice were anesthetized and surgically castrated at 10–12 weeks of age. All experiments were approved by the Institutional Review Committee at Kinki University Faculty of Medicine. Mice were maintained in accordance with institutional guidelines and procedures were carried out in compliance with the standards for use of laboratory animals.

### Pharmacodynamic studies

Pharmacodynamics effects of sorafenib were performed on 20-week-old *PTEN*-mutant mice harboring castration-naïve prostate cancer (CNPC). Sorafenib was administered as a single dose and mice were sacrificed after 24 h. Tumor samples were dissected and processed for western blot analysis.

### Drug chemoprevention studies

Chemoprevention studies were performed on six-week-old *PTEN*-mutant mice. Sorafenib was administered orally by gavage (30 mg/kg, 3X/week) beginning at six weeks of age and was continued until the animals were 15 weeks or 20 weeks of age, at which time the experiment was terminated. The animals were sacrificed and the genitourinary tracts (GUT) were collected *en bloc,* weighed, imaged, and processed for histopathological, immunohistochemical (IHC), and western blot analysis.

### Drug intervention studies

Drug efficacy was determined by performing drug intervention studies on 16-week-old *PTEN*-mutant mice harboring CNPC or CRPC. To induce CRPC, mice were surgically castrated at 10 weeks of age as previously described [30]. Mice were randomized and assigned to treatment groups consisting of control (equal volume of vehicle, 5X/week), sorafenib (gavage, 30 mg/kg, 5X/week), everolimus (oral gavage, 10 mg/kg, 3X/week) or combination sorafenib (gavage, 30 mg/kg, 5X/week) and everolimus (gavage, 10 mg/kg, 3X/week) for 4 weeks. Mice were sacrificed and the GUTs were removed *en bloc*, weighed, imaged and processed for histopathological, IHC, TUNEL and western blot analysis.

### Tumor burden and efficacy determination

Tumor burden was determined by fresh GUT weight or prostate surface area. The surface area of prostate, containing tumor tissue was determined using gross images of fresh GUTs as previously described [30]. Digital images were captured using a Nikon Coolpix 995 digital camera attached to an Olympus SX61 stereomicroscope. The images were spatially calibrated and area measurements were obtained using ImageJ image analysis software [31]. Drug antitumor efficacy was determined by differences in GUTs weight or prostate surface area.

### Histology and immunohistochemical analysis

GUTs were fixed overnight in 10% neutral buffered formalin. Tissues were processed, embedded in paraffin, sectioned and stained with hematoxylin and eosin using standard methods. IHC analysis was performed according to the standard protocols described previously [32]. Assessment of staining was performed visually or using ImageJ analysis software with the Landini color deconvolution plugin V1.5 [33].

### Distribution analysis

Distribution analysis of tumor stroma and epithelial cells compartments was performed on photomicrographs of H&E stained cross sections of the GUT taken at 40X magnification and digitally stitched with Photoshop CS5 Extended (Adobe Systems Inc. San Jose CA). Images were spatially calibrated and digital image masks corresponding to dorsolateral and ventral prostate lobes were painted on. In addition, separate masks corresponding to stroma, normal acini, lgPIN and hgPIN within the dorsolateral and ventral prostates were also generated (Additional file 1: Figure S1). Area measurements corresponding to each individual mask were recorded by the software and exported to a spreadsheet for further statistical analysis.

### Terminal deoxynucleotidyl transferase-mediated dUTP nick endlabeling assay

Terminal deoxynucleotidyl transferase-mediated dUTP nick endlabeling (TUNEL) assays were determined using the *In Situ* Cell Death Detection Kit (Roche Diagnostics Corporation, Indianapolis, IN) according to methods previously described [32].

### Western blot analysis

Protein extraction and immunoblotting were performed as previously described [32]. Semi-quantitative densitometric analyses were assessed using ImageJ analysis software. For all densitometric analyses, protein levels were normalized to GAPDH or total protein.

### Statistical analysis

Data were reported as mean values ± standard error and were statistically analyzed using the Student’s *t*-test for paired analysis and one-way ANOVA for multiple comparisons. *P* < 0.05 was considered statistically significant. Statistical analysis was carried out using Sigmaplot v.13.0 (Systat Software, Inc. San Jose, CA).

## Results

### Pharmacodynamic effects of sorafenib

**Figure 1:**
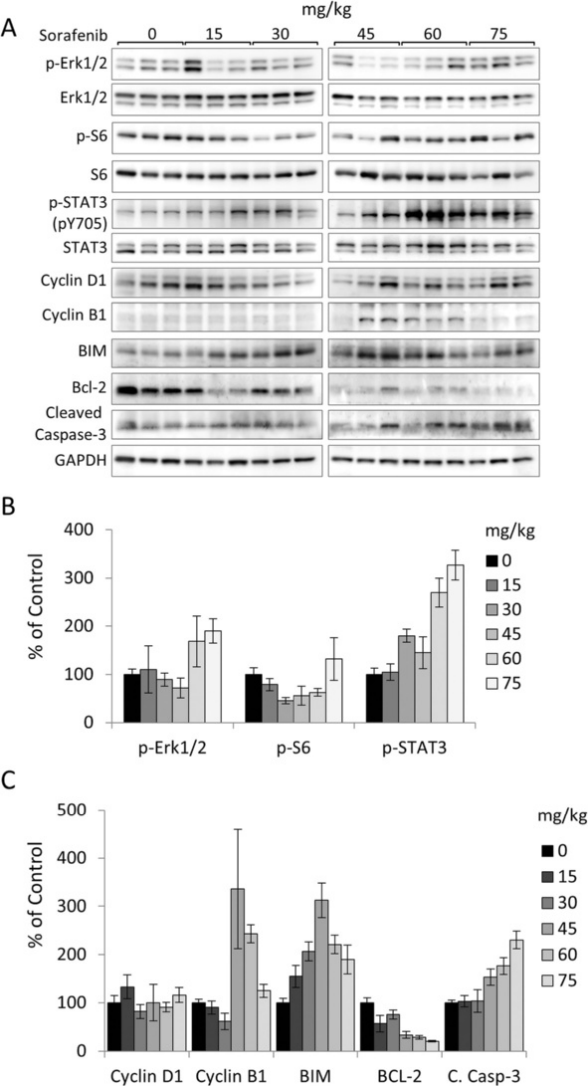
Pharmacodynamic effects of sorafenib administration. Sorafenib was administered at the indicated doses for 24 h to castration-naïve *PTEN*-deficient mice at 20 weeks of age. Prostate tumor lysates were prepared and protein levels were analyzed by western blot. **(A)** Protein levels of phosphorylated and total proteins of ERK, S6 and STAT3, and glyceraldehyde 3-phosphate dehydrogenase (GAPDH). **(B)** Densitometric analysis of p-ERK/ERK, p-S6/S6 and p-STAT3-pY705/STAT3. **(C)** Densitometric analysis of Cyclin D1, Cyclin B1, BIM, Bcl-2 and Cleaved Caspase-3 normalized to GAPDH. Plots are expressed as the mean ± s.e.

In order to establish the preliminary activity of sorafenib in our mouse model and to determine a suitable dose for the intervention studies, we assessed the pharmacodynamic effects of a single dose of sorafenib on the activation of downstream molecules of MAPK, PI3K/AKT/mTOR and JAK/STAT signal transduction pathways, and markers of cellular proliferation and apoptosis. Our findings revealed that for the most part activation of Erk1/2 remained unaffected up to doses of 45 mg/kg, however, levels of phosphorylation increased at 60 and 75 mg/kg (Figure 1A-B). Phosphorylation of S6, a downstream target molecule of the PI3K/AKT/mTOR signaling axis, decreased at doses of 30–60 mg/kg, but increased at the maximum dose of 75 mg/kg (Figure 1 A-B). A dose-dependent activation of STAT3 at tyr705 was also observed (Figure 1A-B). For the most part, expression levels of cyclin D1 were unaffected by a single administration of sorafenib, however, levels of cycling B1 expression were elevated at doses of 45 and 60 mg/kg (Figure 1A,C). Interestingly, there was a dose-dependent effect on the expression levels of pro-apoptotic proteins (BIM and cleaved caspase-3) and decrease of anti-apoptotic Bcl-2 (Figure 1A,C). Based on these findings, we decided to test the efficacy of chronic sorafenib administration at a dose of 30 mg/kg.

### Sorafenib suppresses PTEN-deficient tumor progression

**Figure 2:**
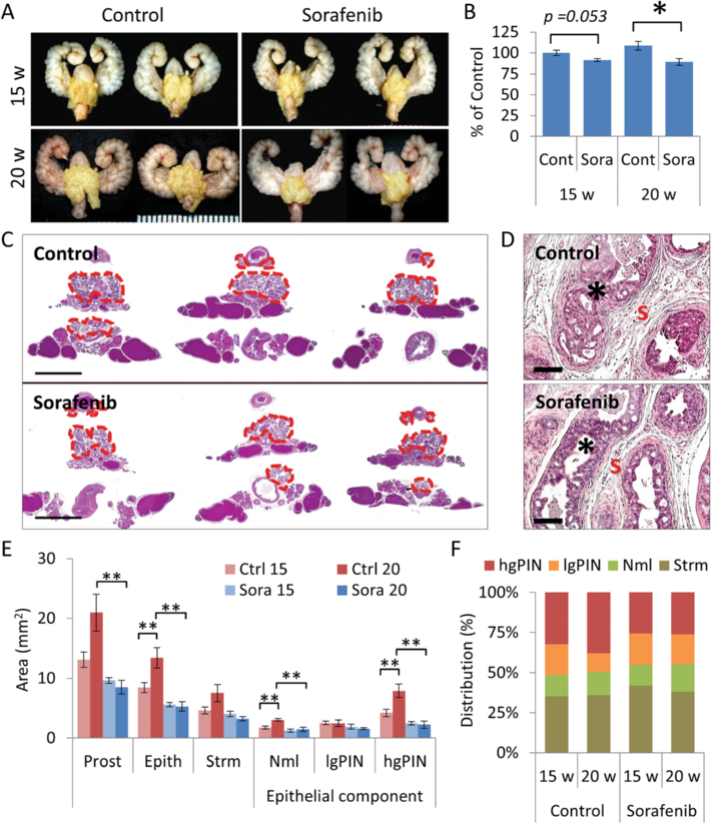
Chemopreventive effects of sorafenib on mouse *PTEN*-deficient prostate hgPIN. Six-week-old *PTEN*-deficient mice were treated with sorafenib (30 mg/kg 3X/week for nine or 14 weeks, *n* = 8 mice/group). **(A)** Representative GUTs from mice after indicated treatment. Prostate tumors are highlighted in yellow. Scale is represented in mm. **(B)** Plots of tumor burden assessed by GUT weight and expressed as the mean ± s.e., *P < 0.05. **(C)** Representative H&E stained tissue sections from mice after indicated treatment. Prostate tumors are delineated by the red dotted line. Scale bars represent 5 mm. **(D)** High magnification images of representative H&E stained tissue sections from mice after indicated treatment. Morphological differences in dysplastic glands (*) and stroma (s) are shown. Scale bars represent 100 μm. **(E)** Plots of absolute tumor tissue distribution analysis expressed as the mean ± s.e., * *P* < 0.05, **P < 0.01. **(F)** Plots of relative tumor tissue distribution analysis expressed as the percent of tumor involvement.

To characterize the antitumor effects of sorafenib *in vivo*, we first sought to determine the chemopreventive effects on *PTEN*-deficient prostate cancer development. In our conditional *PTEN*-knockout mouse model, mice are born cancer-free and develop PIN at eight weeks of age. By 10 weeks of age, half of the mice develop low grade tumors and by 15 weeks 100% of mice develop cancers [30]. From 15 to 20 weeks of age, tumors grow quickly and thus provide an excellent window to assess changes in tumor growth and progression. Therefore, we administered sorafenib orally at a dose of 30 mg/kg 3X/week to cancer-free six-week-old *PTEN*-mutant mice and compared differences in tumor development at 15 and 20 weeks of age. The effects on tumor burden are shown in Figure 2. Compared to vehicle controls, tumor burden based on GUT weight was significantly reduced after treatment with sorafenib in the 20-week-old group (Figure 2A-B). Histologically, tumors from mice treated with sorafenib demonstrated morphological changes in tumor architecture that consisted of decreased reactive stroma and distension of prostatic acini congested with coagulated secretory material (Figure 2C).

To further assess the effects on tumor progression after treatment with sorafenib, we performed histological analysis of the tissue distribution patterns of stromal and glandular tissue within the prostate tumors. This analysis measures differences in tumor development and progression. Control mice showed an increase in the prostate area, consisting of the dorsolateral and ventral prostates between 15 and 20 weeks (Figure 2C). In addition, increases in the stromal and epithelial compartments were also observed. Significant differences between the absolute area measurements of normal and cancerous (hgPIN) glands were observed, but these did not reflect changes in the relative proportions of transformed glands (Figure 2D-E). Notably, mice treated with sorafenib exhibited decreases in the absolute area measurements of the whole prostate, epithelial and stromal compartments, and normal and cancerous glands (Figure 2D). Moreover, marked differences were observed in the relative proportions of the distribution of PIN and cancerous glands in the prostates of control and sorafenib-treated mice at 20 weeks.

**Figure 3:**
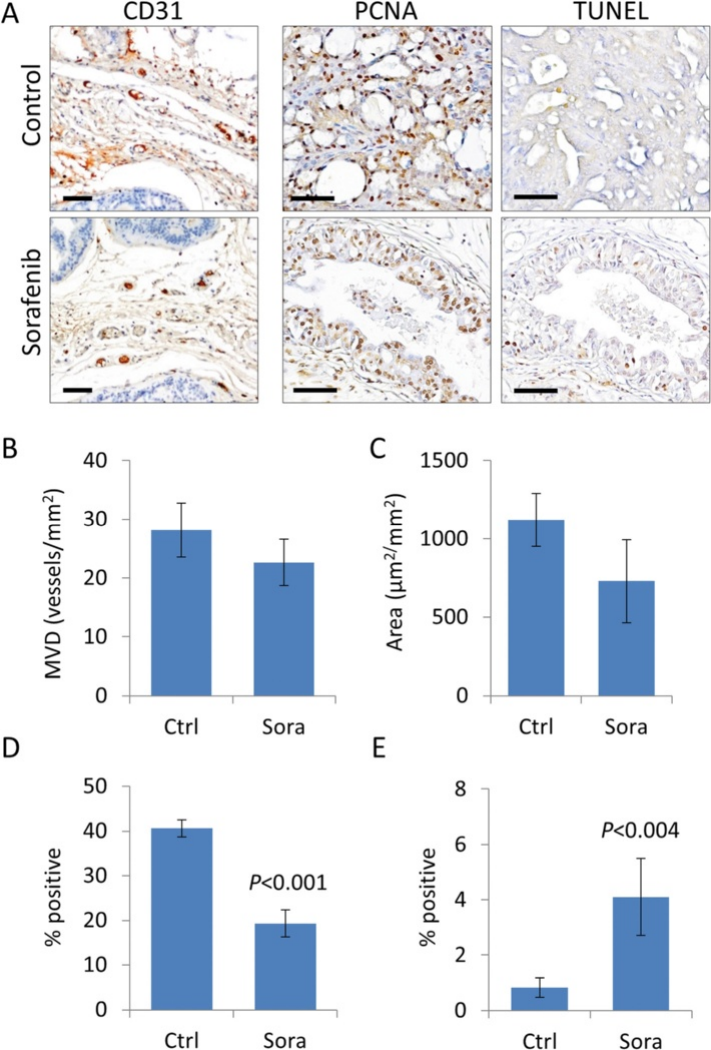
Sorafenib chemoprevention suppresses tumor angiogenesis, proliferation and induces apoptosis. Prostate tumors from 20-week-old control and sorafenib-treated conditional *PTEN*-knockout mice well collected and analyzed to measure MVD and proliferation by IHC for the expression of CD31 and PCNA, respectively, apoptosis by the TUNEL assay. **(A)** Representative CD31 and PCNA immunostained and TUNEL sections of prostate cancer. Positive cells are stained brown. Scale bars represent 100 μm. Plots of MVD **(B)** and microvessel area **(C)** expressed as the mean ± s.e. Plots of cancer cell proliferation rates based on PCNA expression **(D)** and apoptotic rates based on TUNEL positive cells **(E)**. Values are expressed as the mean ± s.e.

We also determined the antiangiogenic effects of treatment with sorafenib in prostate tumors. CD31 was used to analyze microvessel densities (MVD) and microvessel area (MVA). Mice receiving sorafenib for a total of 14 weeks had lower MVD and MVA compared to vehicle-treated controls (Figure 3A-C and Additional file 2: Figure S2). Mice receiving sorafenib also demonstrated significantly lower tumor proliferation (Ki67) rates and induction of apoptosis (TUNEL, Figure 3A,D-E). Altogether, these data show that although sorafenib did not affect the onset of prostate cancer, however, it repressed disease progression through the inhibition of angiogenesis and proliferation, and the induction of apoptosis.

### Therapeutic intervention with sorafenib reduces PCa growth and reverses resistance to everolimus in CRPC

**Figure 4:**
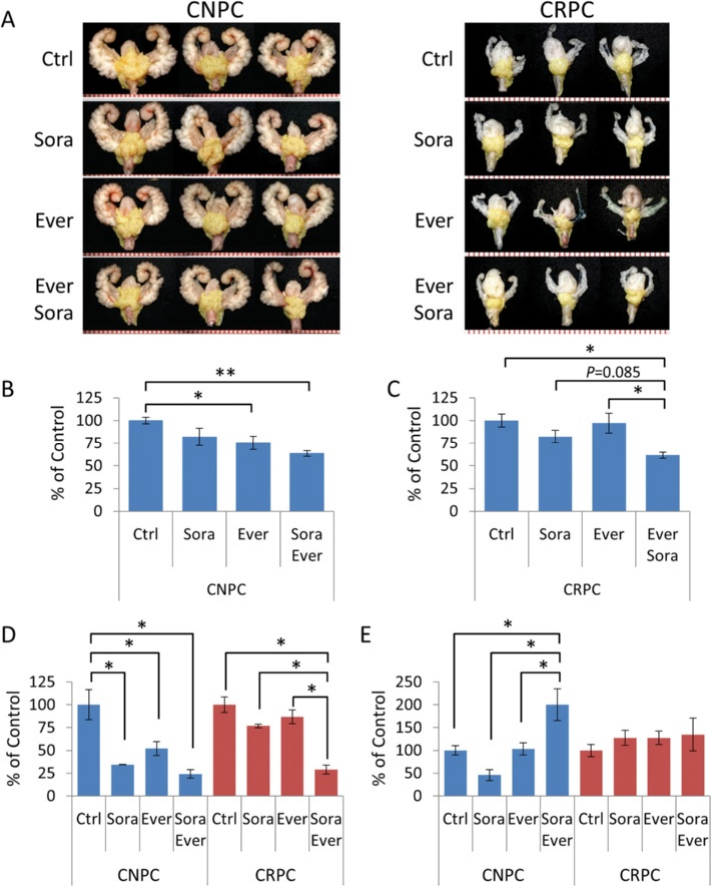
Therapeutic effects of combination therapy with sorafenib and everolimus in *PTEN*-deficient prostate cancer. *PTEN*-deficient mice with CNPC and CRPC were treated with sorafenib, everolimus or sorafenib plus everolimus for 4 weeks as described in the materials and methods (*n* = 8 mice/group). **(A)** Representative images of GUTs from mice after indicated treatment. Prostate tumors are highlighted in yellow. Scale is represented in mm. Plots of tumor burden assessed by tumor surface area for CPNC **(B)** and CRPC **(C)** intervention models. Plots of cellular proliferation rates relative to control mice assessed by the number of Ki67-positive/Ki67-negative cancer cells **(D)** and the cellular apoptotic rates relative to control mice assessed by the number of TUNEL-positive cells bodies/total negative cancer cells **(E)**. All plot values represent the percent of control expressed as the mean ± s.e., *P < 0.05, **P < 0.001.

We next determined the effects of sorafenib on established prostate tumors. Ina addition, we hypothesized that by targeting both the epithelial cancer cells and cells of the tumor microenvironment could potentiate the antitumor effects of sorafenib. Therefore, we evaluated the antitumor effects of treatment for four weeks with sorafenib alone or in combination with the mTOR inhibitor everolimus in 16-week-old mice with *PTEN*-deficient CNPC and CRPC. Treatment effects of the drug interventions, determined by differences in tumor burden measured by tumor area, are shown in Figure 4. An 18% reduction of tumor burden was observed for mice receiving sorafenib monotherapy in both CNPC and CRPC intervention models, however, it was statistically insignificant (Figure 4A-C). In concordance with our previous report [30], monotherapy with everolimus elicited an antitumor effect only in mice with CNPC but not CRPC (Figure 4A-C). Although statistically insignificant (do not test result), the treatment combination of sorafenib and everolimus demonstrated a tendency of augmented tumor burden reductions over monotherapy (18.5% and 11.9% reduction of tumor burden in sorafenib and everolimus treatment groups, respectively) in the CNPC cohort (Figure 4A-B). In the CRPC treatment group, combination therapy overcame therapeutic escape of mTOR monotherapy and provided a modest but statistically insignificant improvement over sorafenib monotherapy (Figure 4A and C).

IHC analysis of Ki67 expression revealed significant suppression of tumor proliferation rates in all mice receiving drug treatments in the CNPC intervention model. Tumor cell proliferation was more strongly inhibited in mice receiving sorafenib compared to everolimus; still, the combination of the two drugs did not augment the antiproliferative effect (Figure 4D). In the CRPC intervention model, only modest reductions in tumor proliferation rates were observed in mice receiving monotherapy with either sorafenib or everolimus, however, combined therapy synergized to enhance the inhibition of tumor cell proliferation (Figure 4D). The apoptotic rate of CNPC tumors treated with combination therapy was significantly greater than that after monotherapy with either sorafenib or everolimus (Figure 4E). In the CRPC model, combination therapy did not result in improved apoptotic rates compared to monotherapy (Figure 4E).

**Figure 5:**
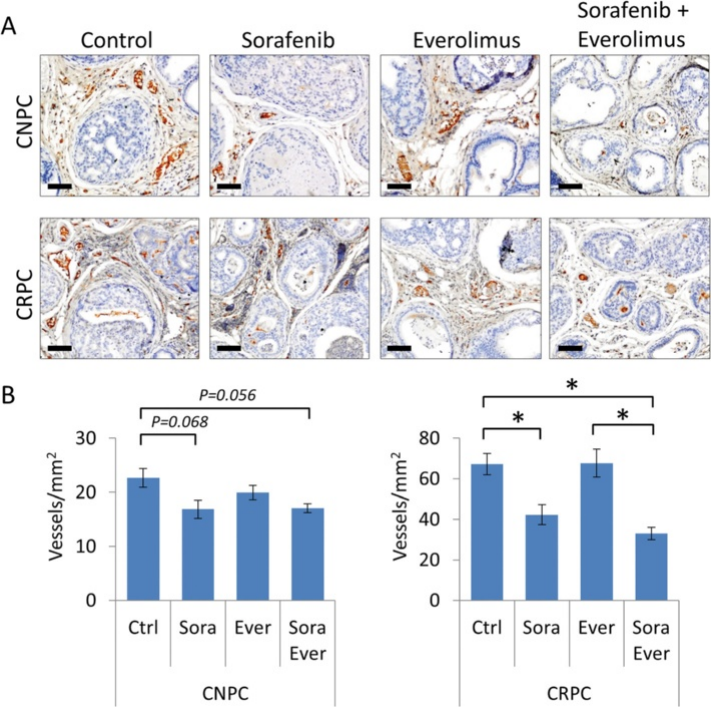
Anti-angiogenic effects of combination therapy with sorafenib and everolimus in *PTEN*-deficient prostate cancer. Assays of MVD assessed in prostate tumors from mice with CNPC and CRPC after drug interventions. **(A)** Representative images showing CD31 immunostaining of prostate tumors after the indicated treatment. Scale bars represent 100 μm. **(B)** Quantitative analysis of MVD according to the presence of CD31-positive microvessels. Values are expressed as the mean ± s.e., *P < 0.05.

We next evaluated the antiangiogenic responses in the tumor stroma after treatment with sorafenib and compared the effects to combination therapy with everolimus. Antiangiogenic responses based on MVD are shown in Figure 5. Treatment with everolimus alone did not affect MVD in either the CNPC or CRPC intervention models. On the other hand, treatment with sorafenib resulted in significant decreases in MVD in both CNPC and CRPC models, however, a higher reduction was observed in the CRPC model (Figure 5A-B). Furthermore, combination therapy did not contribute to enhance the antiangiogenic effects of sorafenib.

### Molecular characterization of major signal pathways in response to combination therapy with sorafenib and everolimus

**Figure 6:**
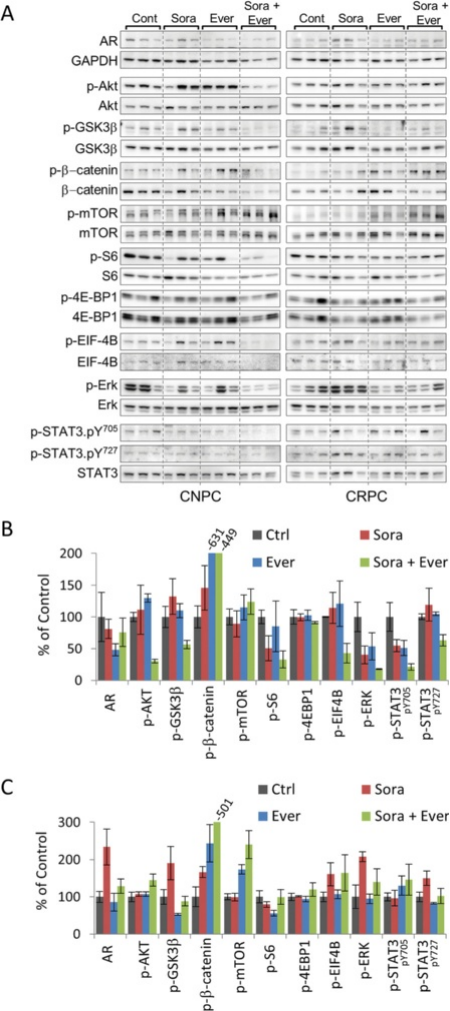
Molecular profiles of *PTEN*-deficient prostate cancer after combination therapy with sorafenib and everolimus. **(A)** Expression of AR, GAPDH, and phosphorylated and total proteins of AKT, GSK3β, β-catenin, mTOR, S6, 4E-BP1, EIF-4B, ERK1/2 and STAT3 in prostate tumor lysates from mice with CNPC and CRPC after the indicated treatments. Densitometric analysis of **(A)** in CNPC **(B)** and CRPC **(C)** intervention models. GAPDH was used as a loading control. Relative protein expression levels were normalized to GAPDH for AR or corresponding total protein for phosphorylated proteins. Plots are expressed as the mean ± s.e. relative to control.

To further characterize the mechanisms by which sorafenib and everolimus function to inhibit tumor growth in *PTEN*-deficient prostate cancer, we investigated the activation of signal transduction pathways associated prostate cancer progression. The expression levels of AR as well as proteins involved in PI3K/AKT, MAPK, and STAT3 signaling pathways were assessed in prostate tissues from mice both CNPC and CRPC intervention experiments (Figure 6). Average levels of AR decreased notably in CNPC mice receiving everolimus, however, mice receiving sorafenib alone or in combination with everolimus experienced marginal reductions (Figure 6A-B). In the CRPC intervention model, mice receiving sorafenib exhibited >2-fold increase in AR expression levels compared to vehicle-treated controls. Monotherapy with everolimus had a minimal suppressive effect on AR expression, and when administered in combination with sorafenib, partially prevented the increase in expression (Figure 6A,C). However, neither treatment decreased the nuclear translocation of AR (Additional file 3: Figure S3)

As expected, levels of AKT phosphorylation increased in response to treatment with everolimus. However, responses to sorafenib varied between individual animals in both CNPC and CRPC intervention models. Interestingly, combination therapy reduced p-AKT levels in the CNPC treatment group, but levels rose in the CRPC group. Notably, phosphorylation of GSK3β in CRPC decreased mice despite elevated levels of phosphorylated AKT suggesting that the combination of so sorafenib and everolimus might cooperate to suppress GSK3 β activity in CRPC. For the most part, other AKT substrates showed similar tendencies of phosphorylation as p-AKT. Treatment with sorafenib alone reduced the levels of Erk1/2 phosphorylation and the effect was further enhanced by the co-administration of everolimus in CNPC (Figure 6A,B). In sharp contrast, mice with CRPC showed a ~2-fold increase in the levels of Erk1/2 phosphorylation after treatment with sorafenib. Relative levels of p-Erk1/2 did not vary with the administration of everolimus. In fact, adding everolimus to sorafenib contributed to reducing the upsurge of Erk1/2 phosphorylation (Figure 6A,C).

We examined the activation of the JAK/STAT3 pathway by analyzing the transcription activation of STAT3 though its phosphorylation at tyrosine 705 (pY705) and serine 727 (pY727) residues. In the CNPC intervention model, levels of constitutively active STAT3-pY705 decreased in response to treatment with sorafenib or everolimus, and combination therapy with both agents markedly reduced transcriptional activity. Overall, the relative levels of STAT3-pY727 phosphorylation were unchanged with monotherapy but were notably reduced with combination therapy (Figure 6A,B). In the CRPC intervention model, treatment with sorafenib did not affect phosphorylation of STAT3-pY705, however, treatment with everolimus alone or in combination with sorafenib tended to increase the expression levels of STAT3-pY705 (Figure 6A,C). Levels of STAT3-pY727 increased in response to treatment with sorafenib. However, this finding was not unexpected since phosphorylation of the serine residue is regulated by the MAPK pathway and the expression pattern of p-STAT3-pY727 paralleled that of p-Erk1/2 (Figure 6A,C).

## Discussion

Prostate tumors are complex structures made up of normal and aberrant epithelial cells that are surrounded by a stromal component composed of fibroblast, endothelial and inflammatory cell populations. This heterogeneous cell mixture provides the structural and metabolic support that allows cancer cells to survive and progress into aggressive phenotypes. The tumor’s stromal vascular network plays a particularly important role since it co-evolves along neoplastic and structural cells. This vascular network, composed of neoangiogenic microvessel, functions in the transport of oxygen, essential nutrients and growth factors to all cell populations within the tumor. In addition, these vascular networks facilitate the physical recruitment of inflammatory cells and the dissemination of metastatic cancer cells. As a result, tumor vasculature has become an important therapeutic target for molecular targeting agents [34]. In this study, we have used a genetically engineered mouse model of *PTEN*-deficient prostate cancer to demonstrate that targeting both dysplastic epithelial cells and the stromal vascular network with sorafenib suppresses tumor growth. Furthermore, we show differences in sorafenib-induced patterns of molecular response between CNPC and CRPC. We also provide preclinical evidence for enhanced antitumor activity of sorafenib when co-administered with everolimus in CRPC.

The mouse model used in this study uses the human *PSA* promoter to drive the conditional inactivation of *PTEN* in the luminal cells of the adult prostate gland [30]. In this manner, normal PTEN expression and function is maintained in other tissue. Inactivation of *PTEN* results in the stage-specific development of prostate cancer that recapitulates many of the features associated with the human disease. Tumors in these mice arise from normal tissues, but follow a multistage process of disease progression. In essence, this provides an excellent window of opportunity to evaluate the chemopreventive effects of potential anticancer agents by evaluating differences in the rates of disease progression. In the present study, pharmacological administration of sorafenib did not alter the onset of cancer, nevertheless, it did suppress tumor growth and progression. The antitumor effects of sorafenib correlated to the inhibition of cell proliferation and the induction of apoptosis in epithelial cancer cells as well as the reduction of MVD and reactive stroma in non-epithelial tumor cells.

The therapeutic effects of sorafenib were also investigated on established CNPC and CRPC. In both intervention models, treatment with sorafenib significantly inhibited cancer cell proliferation and MVD. However, the administration of sorafenib yielded only modest, statistically insignificant therapeutic responses. Interestingly, it was only in the CNPC intervention model that sorafenib induced apoptosis. It is also important to note that sorafenib inhibited p-Erk1/2 in CNPC. Of note, the treatment combination of sorafenib and everolimus inhibited phosphorylation of GSK3β in CRPC mice despite elevated levels of phosphorylated AKT. Nevertheless, phosphorylation of β-catenin increased suggesting that the combination of so sorafenib and everolimus might cooperate to suppress GSK3b activity in CRPC. However, sorafenib elicited the upregulation of phosphorylated Erk1/2 and STAT3-pY727 in CRPC. The activation of MAPK and STAT3 signal transduction pathways has been associated with the abrogation of apoptosis [35,36]. Typically, activation of STAT3 occurs by cytokine mediated JAK phosphorylation of tyrosine 705, however, a second phosphorylation site exists at serine 727 and is phosphorylated through MEK and its transient or constitutive activation in cancer cells has been associated with survival [37,38]. This effect could very well explain why we did not see the induction of apoptosis in CRPC mice treated with sorafenib.

Previous reports have suggested that sorafenib could inhibit prostate cancer cell survival by decreasing proliferation and inducing apoptosis through the downregulating of AR. However, in our study, treatment with sorafenib failed to downregulate AR expression *in vivo*. In fact, treatment with sorafenib increased AR expression in CRPC. This effect could be attributed to the increased levels of MAPK and STAT3 pathway activation resulting from sorafenib administration especially since both MAPK and STAT3 signal pathways are associated with the ligand independent activation of AR in CRPC [39,40]. Still, the clinical efficacy of sorafenib monotherapy for advance prostate cancer has been limited despite favorable outcomes in other cancer types such as kidney and liver [41,42]. We believe that the findings from our study may shed some light for the shortcomings of sorafenib monotherapy in treating human CRPC.

Recent reports have shown that the treatment combination of sorafenib and mTOR inhibitors result in a greater antitumor effect. [24,26-28]. Therefore, we hypothesized that by using the combination of sorafenib and everolimus, we could target the tumor microenvironment as well as direct cancer cell survival and compensatory pathways that could possibly restore treatment responses of CRPC. In both CNPC and CRPC intervention models, combination therapy demonstrated a clear tendency of improved tumor suppression. More importantly, the treatment combination of sorafenib and everolimus overcame therapeutic escape from single agent therapy in CRPC.

## Conclusions

In summary, we have utilized a genetically engineered mouse model of prostate cancer to demonstrate differential effects of treatment with sorafenib in *PTEN*-deficient CNCP and CRPC and provide mechanistic insights into the molecular responses. Finally, we provide preclinical evidence for the development of targeted treatment strategies based on the use of multikinase inhibitors, such as sorafenib, in combination with mTOR inhibitors for the treatment of advanced prostate cancer.

## Additional files

Additional file 1: Figure S1. Method for tissue distribution analysis in mouse prostate cancer. (A) Representative image of an H&E stained cross section of mouse genitourinary tract (GUT). (B) Same cross section with digital masks added corresponding to the various histological features of the mouse prostate. Additional file 2: Figure S2. Analysis of CD31 expression by western blot in prostate tumor lysates from 20-week-old control and sorafenib-treated conditional *PTEN*-knockout mice described in Figure 3. GAPDH was used as a loading control. Additional file 3: Figure S3. Androgen receptor expression in tumors from *PTEN*-deficient knockout mice. Immunohistochemical analysis of the androgen receptor in castration-naive prostate cancer (CNPC) (A) and castration-resistant prostate cancer (CRPC) (B) drug intervention models described in Figure 4. Scale bars represent 100 μm.
