# Supplementary figures and images for: Evaluation of in vivo responses of sorafenib therapy in a preclinical mouse model of PTEN-deficient of prostate cancer
Source: J Transl Med. 2015 May 8;13:150. doi: 10.1186/s12967-015-0509-x (PMC4438623; doi:10.1186/s12967-015-0509-x)

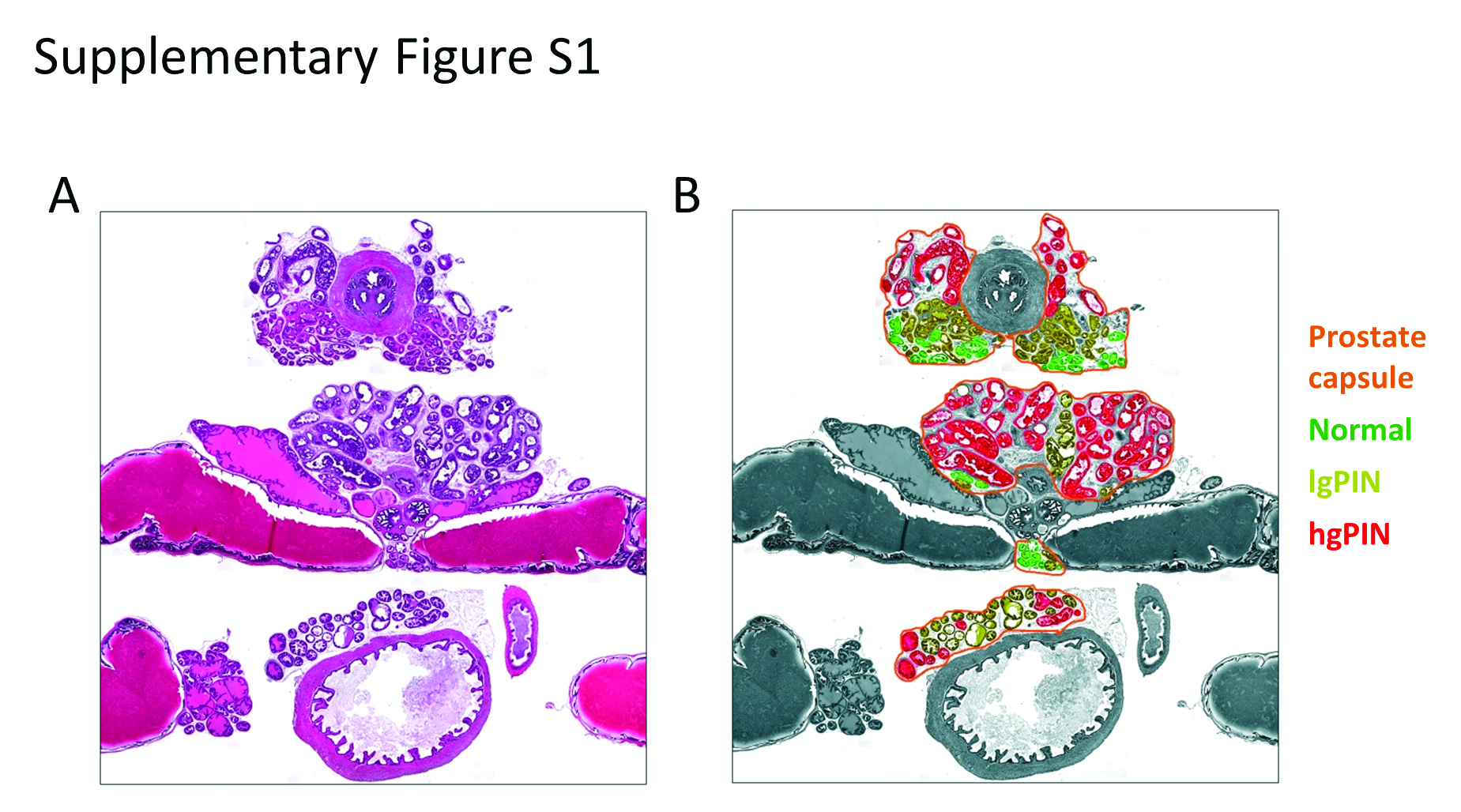

Supplement: Additional file 1: Figure S1. — Method for tissue distribution analysis in mouse prostate cancer. (A) Representative image of an H&E stained cross section of mouse genitourinary tract (GUT). (B) Same cross section with digital masks added corresponding to the various histological features of the mouse prostate. [file 12967_2015_509_MOESM1_ESM.tiff]

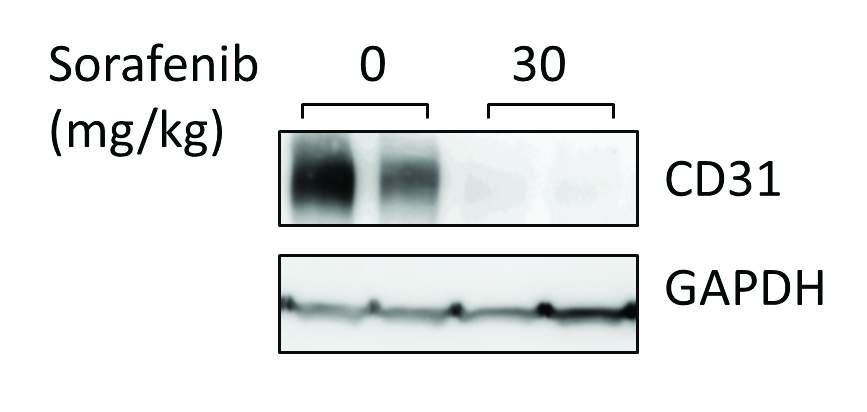

Supplement: Additional file 2: Figure S2. — Analysis of CD31 expression by western blot in prostate tumor lysates from 20-week-old control and sorafenib-treated conditional PTEN-knockout mice described in Figure 3. GAPDH was used as a loading control. [file 12967_2015_509_MOESM2_ESM.tiff]

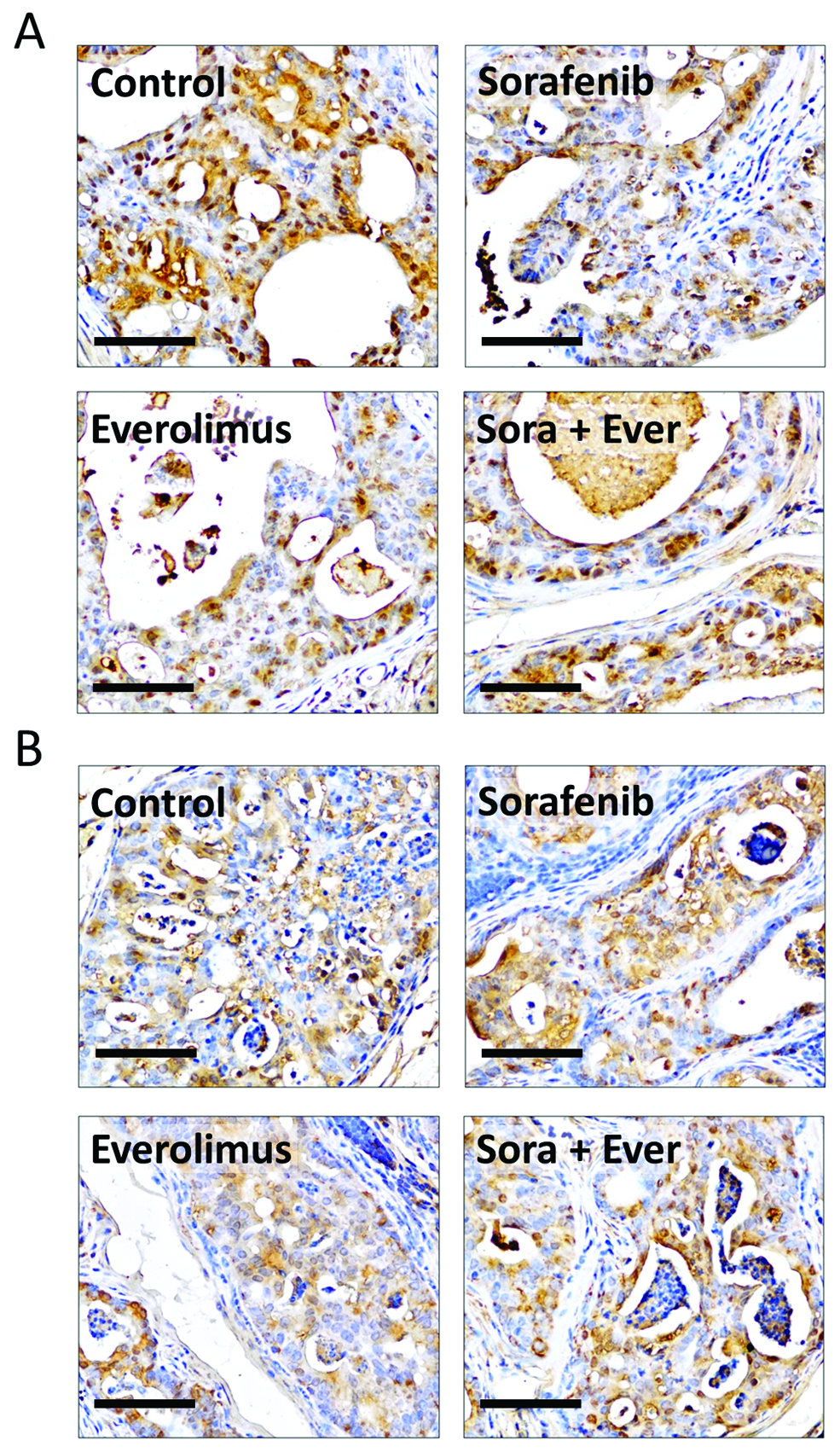

Supplement: Additional file 3: Figure S3. — Androgen receptor expression in tumors from PTEN-deficient knockout mice. Immunohistochemical analysis of the androgen receptor in castration-naive prostate cancer (CNPC) (A) and castration-resistant prostate cancer (CRPC) (B) drug intervention models described in Figure 4. Scale bars represent 100 μm. [file 12967_2015_509_MOESM3_ESM.tiff]
